# Molecular Characterization of Influenza A/H3N2 Virus Isolated from Indonesian Hajj and Umrah Pilgrims 2013 to 2014

**DOI:** 10.3390/life13051100

**Published:** 2023-04-27

**Authors:** Agustiningsih Agustiningsih, Irene Lorinda Indalao, Krisnanur A. Pangesti, Caecilia H. C. Sukowati, Ririn Ramadhany

**Affiliations:** 1 Eijkman Research Center for Molecular Biology, National Research and Innovation Agency of Indonesia (BRIN), B.J. Habibie Building, Jl. M.H. Thamrin No. 8, Jakarta Pusat, DKI, Jakarta 10340, Indonesia; 2 Ministry of Health of the Republic of Indonesia, Jl. H.R. Rasuna Said Blok X.5 Kav. 4-9, Jakarta Selatan, DKI, Jakarta 12950, Indonesia; 3 Fondazione Italiana Fegato ONLUS, AREA Science Park, Basovizza, 34049 Trieste, Italy

**Keywords:** Hajj pilgrim, influenza A/H3N2 virus, HA gene, NA gene, vaccine

## Abstract

The Hajj and Umrah are the annual mass gatherings of Muslims in Saudi Arabia and increase the transmission risk of acute respiratory infection. This study describes influenza infection among pilgrims upon arrival in Indonesia and the genetic characterization of imported influenza A/H3N2 virus. In total, 251 swab samples with influenza-like illness were tested using real-time RT-PCR for Middle East Respiratory Syndrome Coronavirus (MERS-CoV) and influenza viruses. Complete sequences of influenza A/H3N2 HA and NA genes were obtained using DNA sequencing and plotted to amino acid and antigenicity changes. Phylogenetic analysis was performed using a neighbour-joining method including the WHO vaccine strains and influenza A/H3N2 as references. The real-time RT-PCR test detected 100 (39.5%) samples positive with influenza with no positivity of MERS-CoV. Mutations in the HA gene were mainly located within the antigenic sites A, B, and D, while for the NA gene, no mutations related to oseltamivir resistance were observed. Phylogenetic analysis revealed that these viruses grouped together with clades 3C.2 and 3C.3; however, they were not closely grouped with the WHO-recommended vaccine (clades 3C.1). Sequences obtained from Hajj and Umrah pilgrims were also not grouped together with viruses from Middle East countries but clustered according to years of collection. This implies that the influenza A/H3N2 virus mutates continually across time.

## 1. Introduction

The Hajj pilgrimage is the biggest annual mass gathering, where nearly 2–3 million Muslims from more than 180 countries around the globe assemble in Mecca, Kingdom of Saudi Arabia (KSA) [1]. Muslims worldwide are all obliged to attend the Hajj at least once in their lifetime at a specific time, while in contrast, Umrah is not compulsory and can be performed at any time of the year. The overcrowding of pilgrims from different parts of the world within a confined area during the Hajj and Umrah can increase the risk of infectious disease transmission, especially acute respiratory infections [2]. A previous study has reported that pilgrims were likely to develop respiratory symptoms during Hajj. Furthermore, the pilgrims who acquire respiratory viruses during the Hajj can spread the infection in their home countries upon their return [3,4].

The emergence of Middle East Respiratory Syndrome Coronavirus (MERS-CoV) in Middle East countries has raised global awareness concerning transmission among Hajj pilgrims [5]. Until January 2023, 2603 laboratory-confirmed cases of MERS-CoV with 935 deaths were reported to the WHO (a case fatality ratio of 36%) [6]. The Government of the Republic of Indonesia, through the Ministry of Health, gives special attention to the Hajj pilgrimage, since Indonesia, the world’s largest Muslim country, sends large numbers of pilgrims, which are allowed by the Government of KSA, to the Hajj annually. Each person who acquired acute severe respiratory infections and was admitted to hospitals upon arrival from the Hajj pilgrimage was tested for MERS-CoV using real time Reverse Transcription-Polymerase Chain Reaction (RT-PCR) [7].

Several studies conducted after the emergence of MERS-CoV reported that MERS-CoV was not detected among Hajj pilgrims; however, the influenza virus was the most common virus isolated from symptomatic patients during Hajj. Other respiratory viruses such as rhinovirus, adenovirus, respiratory syncytial virus, and human metapneumovirus were also detected [3,8,9,10].

Influenza viruses are one of the most common air-borne infectious pathogens. These viruses belong to the Orthomyxoviridae family that includes three genera, the influenza A, B, and C. Of those three groups of viruses, the influenza A virus is the most virulent and is associated with annual epidemics, zoonotic influenza, and global pandemic with high mortality and morbidity rates [11]. Three devastating pandemics in the twentieth century were all caused by the influenza A virus. The first pandemic was the 1918 Spanish Flu caused by the A/H1N1 subtype, followed by the 1957 Asian flu caused by the A/H2N2 subtype, and the 1968 Hong Kong flu caused by the A/H3N2 subtype. Annually, a million cases of illness with mild-to-severe respiratory syndromes and approximately half a million deaths from across region were reported to be associated with seasonal influenza epidemic [11,12].

Influenza viruses that commonly infect and cause illness in humans during winter months in temperate countries, also known as seasonal influenza, are influenza A subtype H1N1pdm, influenza A subtype H3N2, and influenza B viruses [12]. Among influenza A subtypes, the H3N2 subtype was known to have robust antigenic drift, which creates high heterogeneity over time [13]. This subtype was also reported to cause more severe illness and outbreaks than the H1N1 subtype and influenza B viruses [14]. Influenza virus surface glycoproteins, the Hemagglutinin (HA) and Neuraminidase (NA), are the target of the host immune system, and therefore have frequent mutations [15,16,17]. The mutations events that commonly occur within the genome of influenza viruses raise the concern of the changing characteristics of influenza viruses obtained from travellers, particularly the Hajj and Umrah pilgrims.

Mass gathering during Hajj and Umrah can increase the risk of transmission of different strains of influenza A/H3N2 virus, which are then imported to Indonesia; however, limited data are available concerning the influenza characterization from Hajj and Umrah pilgrims that travelled back to Indonesia. Therefore, studying influenza virus characteristics from Indonesian pilgrims is considered necessary to monitor this possibility, as numerous Muslims from Indonesia travel to KSA annually during Hajj season. This study aimed to investigate the molecular characteristics of the influenza A/H3N2 virus from Hajj and Umrah pilgrims in Indonesia from 2013 to 2014 who developed respiratory infections after travelling from the Kingdom of Saudi Arabia.

## 2. Materials and Methods

### 2.1. Ethics

This study was a descriptive study using laboratory experiments for confirmation. The ethical consideration of this study was approved by the ethical committee of the National Institute of Health Research and Development (NIHRD) of the Ministry of Health of Republic Indonesia, no. LB.02.01/5.2/KE.082/2015. Clinical samples obtained in this study were part of NIHRD surveillance for MERS-CoV as the new emerging and re-emerging diseases preparedness program.

### 2.2. Population Study and Sampling Strategy

The study samples were 251 clinical specimens of nasal swabs, tracheal swabs, or sputum of Indonesian Hajj and Umrah pilgrims with influenza-like illness (ILI) symptoms from October 2013 to December 2014. These samples were sent from different hospitals or District Health offices located throughout the Provinces in Indonesia (Sumatra, Java, Bali, Sulawesi, and Kalimantan Islands) to NIHRD as the part of surveillance program for MERS-CoV in Hajj and Umrah pilgrims. All samples were resuspended in Hank’s Balanced Salt Solution (HBSS) transport medium and sent directly for further real time RT-PCR testing in NIHRD, Jakarta.

### 2.3. RNA Isolation and Confirmatory Real-Time RT-PCR for MERS-CoV and Influenza Virus

Viral RNA was extracted directly from 140 µL of the clinical sample using QIAmp Viral RNA Mini Kit (Qiagen, Hilden, Germany), according to the manufacturer’s instruction. The MERS-CoV examination was performed using real-time RT-PCR following WHO interim guidance [7,18,19]. Influenza A and B virus detection and subtyping for seasonal influenza A virus were performed using real-time RT-PCR following the WHO protocol [20]. These assays are based on TaqMan chemistry and include a panel of oligonucleotide primers together with dual-labelled hydrolysis probe to detect MERS-CoV, influenza A, influenza B, H1pdm09, and H3. All tests were conducted using SuperScript^TM^ III Platinum Taq One-Step qRT-PCR Kit (Invitrogen, Carlsbad, CA, USA) in BioRad CFX96 Touch Real-Time PCR Detection System (BioRad Laboratories, Hercules, CA, USA).

### 2.4. Direct Sequencing of Influenza A/H3N2 Virus

All samples confirmed as positive for influenza A/H3N2 virus were selected for molecular analysis. The reverse transcription and amplification of complete coding DNA sequence (CDS) of HA and NA genes were conducted according to previous study [21]. Direct sequencing was performed using PCR products purified by QIAquickTM PCR Purification Kit (QIAGEN, Hilden, Germany), followed by DNA sequencing using Big Dye Terminator V.3.1 Cycle Sequencing Ready Reaction Kit (Applied Biosystem, Foster City, CA, USA), according to the manufacturer’s instruction. After purification using Big Dye X Terminator Purification Kit (Applied Biosystem, Foster City, CA, USA), the reactions were resolved on an automatic sequencer ABI-Prism 3130xl Genetic Analyzer (Applied Biosystems 337 DNA, Perkin Elmer, Waltham, MA, USA). Finally, the nucleotide sequences were edited and assembled using Sequencer version 5.2.4.

### 2.5. Phylogenetic Analysis of Influenza A/H3N2 Virus

Phylogenetic tree was generated using software MEGA 6 [22] with neighbour-joining algorithm and 1000 replications. WHO vaccine strains and other sequences from other countries were included in the analysis. The sequences of WHO vaccine strains ranging from 2010 to 2022 included in the analysis were as follows: A/Perth/16/2009, A/Victoria/361/2011, A/Texas/50/2012, A/Switzerland/9715293/2013, A/Hong Kong/4801/2014, A/Singapore/INFIMH-16-0019/2016, A/Kansas/14/2017, A/Hong Kong/45/2019, A/Cambodia/E0826360/2020, and A/Darwin/9/2021. In addition, the H3N2 consensus sequences were kindly provided by Influenza Division, Centers for Disease Control and Prevention, Atlanta, GA, USA.

## 3. Results

### 3.1. MERS-CoV and Influenza Virus Confirmatory

We analysed 251 swab samples obtained from Hajj and Umrah pilgrims admitted to hospitals with ILI from 5 islands in Indonesia, Java, Sumatra, Bali, Sulawesi, and Kalimantan. These patients mainly originated from Java Island (53.7%), followed by patients from Sumatra Island (26.7%). The mean age of all patients was 57.4 ± 16.6 years old, with a comparable male (n = 126) vs. female (n = 125) ratio (Table 1).

Real-time RT-PCR confirmed that MERS-CoV was not detected (0%) in all 251 patients. All samples were then tested for influenza virus, in which 100 cases (39.8%) were positive. From these positive cases, viral subtyping showed that 15 cases (6%) were influenza A/H3N2, 51 cases (20.3%) were A/H1N1pdm, 4 cases (1.6%) were influenza A non H1pdm/H3/H5 (unsubtype), 29 cases (11.5%) were influenza B, and 1 (0.4%) case was a double infection of influenza A/H1pdm and influenza B. The distribution of the influenza viral subtypes among the grouping parameters (sex, age, and origin) is described in Table 1.

For age parameter, patients were categorized as lower than, equal to 50 and above 50 years old, since above 50 years or older is the high-risk group and prone to severe influenza infection. From all samples, the majority of patients reported to have respiratory illness were above 50 years old (70.1%). Although most of them were not infected by influenza viruses, 74 out of 176 patients aged above 50 had influenza infection, while 23 out of 42 patients aged less than 50 were infected with influenza viruses. As predicted, patients over 50 years of age were more likely to test positive for infection with virus influenza A compared to younger patients (75% vs. 25%). Other aetiologies that caused respiratory illness in negative MERS-CoV and influenza patients were not explored in this study.

### 3.2. Influenza A/H3N2 Virus Analysis

This study further focused on the patients with an infection of influenza A/H3N2 virus. Clinically, the infection of influenza A/H3N2 was more severe than A/H1N1 or B in terms of fever, leukopenia, and C-reactive protein [14].

The detailed data of fifteen samples of positive influenza A/H3N2 cases, such as clinical symptoms, were traced back from the patients’ database. The majority of patients had fever ≥ 38 °C (83.3%), cough (75.0%), sore throat (41.7%), and dyspnoea (75.0%). From these 15 samples, an amplification of the CDS of HA and NA genes was obtained from 8 samples with a length of 1701 and 1410 base pairs, respectively. The HA and NA sequences have been deposited in the Global Initiative on Sharing All Influenza Data (GISAID) with accession number EPI2274939-EPI2274954.

**Figure 1:**
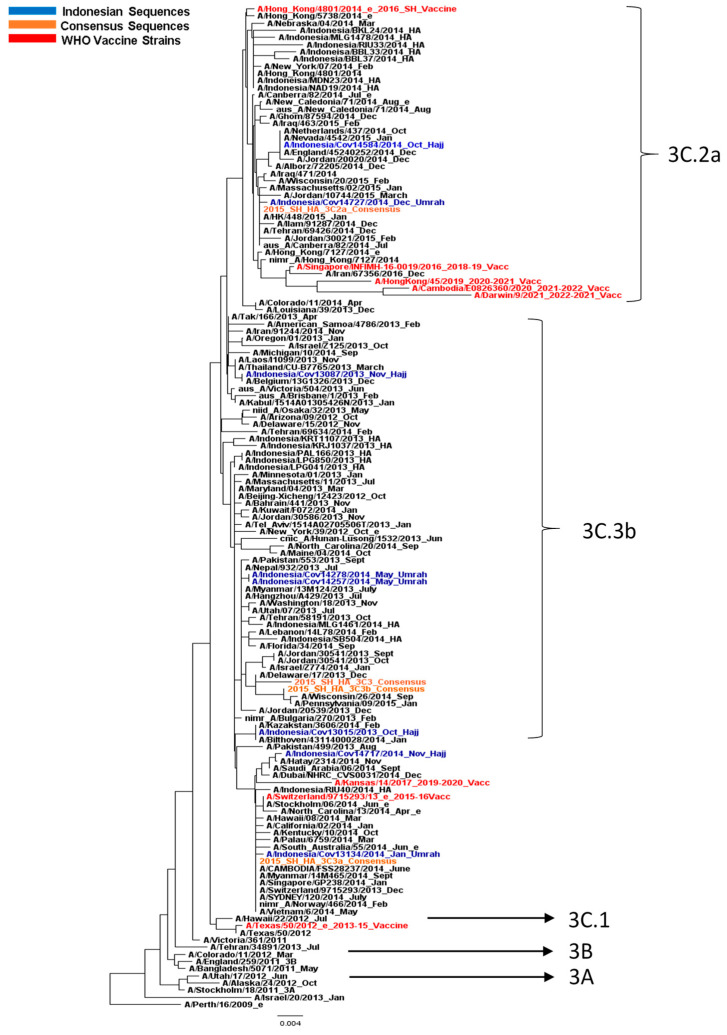
Phylogenetic tree of HA gene of H3N2 virus. Indonesian sequences obtained from Hajj and Umrah pilgrims are in blue, consensus sequences are in orange, and the WHO vaccine strains are in red.

The DNA sequences were then subjected to the construction of phylogenetic trees. In general, the HA phylogenetic tree depicted the continuous changes in the influenza A/H3N2 virus according to the time when major Indonesian and global circulating viruses in 2013 and 2014 clustered into clades 3C.2 and 3C.3. The HA phylogenetic tree showed typical ‘ladder-like’ phylogeny, with the replacement of old strains by the newer one. Interestingly, the WHO vaccine strains A/Switzerland/9715293/2013 and A/Hong Kong/4801/2014, the vaccine strains for the 2015 to 2016 influenza season, were grouped within these clades (Figure 1). Instead of being clustered together with the respective 2013–2015 vaccine strain in clade 3C.1 (A/Texas/50/2012), the HA phylogenetic analysis grouped the Indonesian pilgrim’s sequences into clades 3C.2 and 3C.3 (3C.3a, 3C.2a, 3C3b). The Indonesian sequences did not cluster closely with sequences that originated from Middle East countries. Interestingly, HA sequences obtained from Hajj (clade 3C.3) were separated from Umrah pilgrims (3C.2). It should be noted that the dates of departure of these pilgrims were different, as the sequences were collected from October 2013 to December 2014.

**Figure 2:**
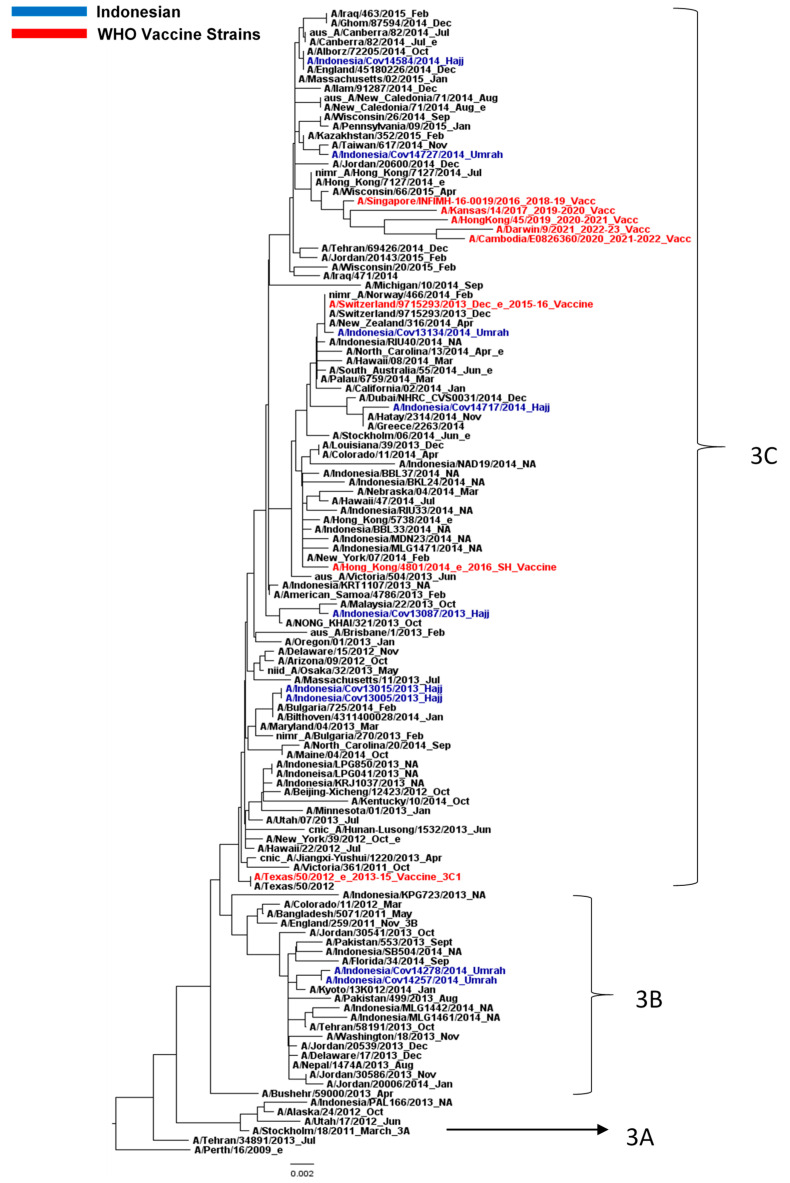
Phylogenetic tree of NA gene of H3N2 virus. Indonesian sequences obtained from Hajj and Umrah pilgrims are in blue, while the WHO vaccine strains are in red.

The phylogenetic tree of the NA gene (Figure 2) showed a different structure from that of the HA. Six and two samples were grouped in clades 3C and 3B, respectively, with no distinction between Hajj and Umrah clustering. These data were expected since the evolution between HA and NA genes were dissimilar, although both HA and NA are the surface glycoproteins. Simplified phylogenetic trees of HA (Figure S1) and NA (Figure S2) gene, completed with their bootstrap values, were provided in Supplementary Data.

### 3.3. Amino Acid Analysis of HA and NA

To assess whether the DNA sequences of the HA and NA genes of these patients correlated with the given vaccines, the residues analysis in the HA amino acid sequences, particularly in the HA1 domain, using H3 numbering against vaccine strains in the 2013–2015 season was conducted. This was to understand the discrepancy of the separate clustering of the samples to the vaccine strain in the 2013 to 2015 influenza season.

As shown in Table 2, mutations within the HA gene were mainly located within the antigenic sites A, B, and D compared to the WHO vaccine strain for 2013–2015 (A/Texas/50/2012). We noticed that all samples (8/8) showed variations of N145S, V186G, P198S, and F219S. Referring to data in the literature, these mutations are the signature of clades 3C.2 and 3C.3 [23]. Furthermore, amino acid changes were not related to the place of origin of the pilgrims. These findings emphasized that multiple mutations occurred within the HA gene across time.

**Figure 3:**
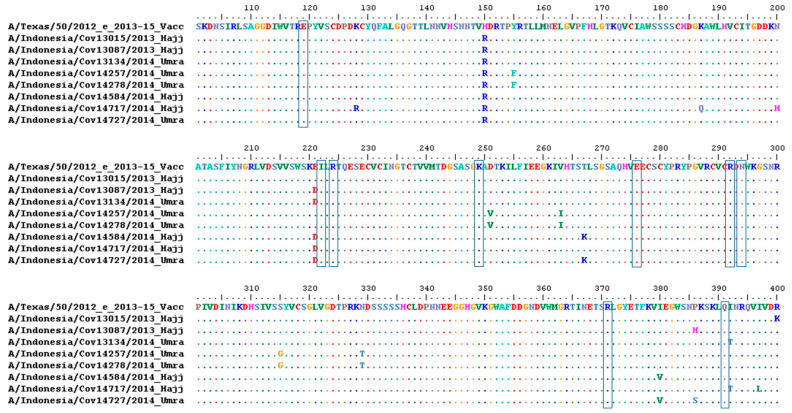
Amino acid alignment of the neuraminidase (NA) from Indonesian pilgrimages compared to WHO vaccine strain for 2013–2015 influenza season—A/Texas/50/2012. The blue square highlights the position of the genetic marker of oseltamivir resistance; and amino acid position 119, 222, 224, deletion 245–248, deletion 247–250, 249, 276, 292, 294, 371, and 391 (N2 numbering system) [29].

For NA residues analysis, in terms of the genetic marker of antiviral susceptibility, specific mutations in the NA conferring the resistance to oseltamivir based on a previous study, which are E119D, E119I, E119V, I222L, R224K, deletion 245–248, deletion 247–250, K249E, E276D, R292K, N294S, R371K, and Q391K [28,29], were not found in this study (Figure 3). We also found no mutations in the catalytic site of NA residues R118, D151, R152, R224, E276, R292, R371, and Y406. These residues are commonly conserved in influenza A and B; therefore, these sites are known as antiviral targets that block NA activity [28].

## 4. Discussion

Numerous Indonesian pilgrims travel to KSA to perform the Hajj and Umrah pilgrimages each year. According to the Ministry of Religion Affairs, Republic of Indonesia, approximately 150,000 pilgrims from Indonesia travelled to KSA in 2013 and 2014 [30], joining other pilgrims from around the world [31]. The total number of Indonesian pilgrims travelling to the KSA was greater, as another pilgrimage called Umrah was conducted outside the Hajj season. Even though the pilgrims gathering during Umrah were not as crowded as during the Hajj period, the acute respiratory infection may easily transmit from and to other pilgrims due to overcrowding. It was reported that among patients returning from pilgrimages, respiratory infection was the fourth leading cause of death, following cerebral vascular, diabetes, and cardiovascular diseases [32]. In parallel, respiratory infection was the second leading aetiology reported among Hajj pilgrims from Indonesia [33].

To the best of our knowledge, this study is the first in Indonesia that focuses on influenza infection and also the genetic characterization of the surface glycoprotein of influenza A/H3N2 virus obtained from Hajj and Umrah pilgrims. As a part of the new emerging and re-emerging diseases preparedness program, the Ministry of Health of Indonesia, which includes the NIHRD, conducted surveillance of MERS-CoV among Indonesian pilgrims that arrived from KSA and showed ILI symptoms after Hajj or Umrah pilgrimage. In this study, we found no MERS-CoV infection among the samples tested. A similar finding was also reported in another study involving a larger number of samples obtained from Indonesian Hajj pilgrims with febrile and respiratory symptoms [34]. As for the prominent viral aetiology of respiratory infection among Hajj pilgrims, influenza viruses were reported from French and Austrian pilgrims [8,35], although another report detected human rhinovirus among hospitalized patients during Hajj season [10].

Based on the date of onset data available from seven cases of influenza A/H3N2 infection, four cases showed clinical symptoms one to two days before departure from KSA, while three cases showed clinical symptoms after arrival in Indonesia. This data enhanced the possibility of the pilgrims acquiring the influenza virus in KSA, since influenza virus has an incubation time of approximately seven days. The most common clinical symptom documented in the Hajj and Umrah Pilgrims was fever, which is the most common symptom in ILI patients.

Performing Hajj is obligatory for Muslims, as Hajj is one of the five pillars of Islam. Many Muslims in Indonesia wait decades for the opportunity to perform Hajj due to its high cost and limitations to the total pilgrims allowed to enter KSA during Hajj season. By the time Indonesian pilgrims have the opportunity, the majority of them are old and may have a multitude of age-related health concerns [33]. As we observed in this present study, the highest number of pilgrims admitted to hospitals with ILI symptoms comprised the elderly, who are more likely to have health comorbidities and a proneness to become sick. This finding also highlights the need for testing other aetiologies that cause respiratory infections, since 60% of cases in this study were negative for MERS-CoV and influenza viruses.

Influenza A virus is a negative-sense enveloped RNA virus with a whole-genome size that is approximately 13.5 kb in length. The structure of the genome consists of 8 segmented single-stranded RNA genes: polymerase basic 1 (PB1), polymerase basic 2 (PB2), polymerase acidic (PA), nucleoprotein (NP), matrix (M), nonstructural (NS), and HA and NA [11]. The HA and NA genes encoded the HA and NA surface glycoproteins mainly become the basis of influenza A subtype classifications. There are currently 18 HA subtypes and 11 NA subtypes, most of which circulate in wild birds, but only 3 combinations are known to have circulated widely in humans: A/H1N1, A/H2N2, and A/H3N2. Of these, the A/H1N1 and A/H3N2 subtype viruses currently cause seasonal influenza virus epidemics [36].

To understand the genetic origin of influenza from these patients, we performed a phylogenetic tree analysis of the CDS of the HA and NA genes. Phylogenetic analysis showed that Indonesian influenza A/H3N2 isolates were not exclusively clustered together with viruses from Middle East countries but grouped closely with the sequences from other countries according to years, suggesting these viruses shared similar molecular characteristics. The phylogenetic analysis in this study showed the global circulation of the influenza A/H3N2 virus [37]. The potential for the pathogen transmission of airborne diseases including influenza virus has increased because air travel activities worldwide have risen significantly, connecting more people between islands or continents [38,39].

The phylogenetic tree of the HA gene classified the H3N2 virus into genetic clades 3C.2 and 3C.3 (3C.2a, 3C.3a and 3C.3b), which circulated worldwide, including in Indonesia. In this study, the Indonesian pilgrims’ sequences were grouped into clades 3C.2 and 3C.3, the different clades that the WHO recommended vaccines for in the 2013 to 2015 influenza season: A/Texas/50/2012 (clade 3C.1). Interestingly, none of the Indonesian pilgrims’ sequences collected in 2013–2014 clustered in this clade, suggesting distinct antigenic characteristics between the vaccine strain for 2013–2015 season and the Indonesian virus. This finding also highlights the importance of evaluating whether the vaccine given to the Hajj and Umrah pilgrims is still appropriate [40]. The changing circulating clades are the reason for continuously monitoring and updating the WHO-recommended vaccine. A/Texas/50/2012 was replaced with A/Switzerland/9715293/2013, the WHO vaccine strain classified as clade 3C.3 for the 2015 to 2016 influenza season. However, as clade 3C.2 had majorly circulated and then replaced clade 3C.3, the WHO substituted the vaccine strain A/Switzerland/9715293/2013 for A/Hongkong/4801/2014 for the 2016 influenza season.

Compared to NA, which is relatively stable, the amino acids of HA mutates frequently in order to escape host antibody recognition [41]. The A to E antigenic sites within the HA1 domain are recognized to be the target of neutralizing antibodies, causing these sites to be under the selective pressure of the human immune system. This domain was identified to have mutated to escape the human immune system, resulting in antigenic changes over time, which is commonly known as antigenic drift [42]. An antigenic drift of the H3N2 virus had occurred several times and has been well documented. WHO-recommended vaccine strains were replaced regularly to match the circulating H3N2 global circulating strain.

NA protein, by its enzymatic activity, facilitates virion releases from host cells. The catalytic site of the NA is constituted of eight functional residues (R118, D151, R152, R224, E276, R292, R371, and Y406), surrounded by eleven framework residues (E119, R156, W178, S179, D198, I222, E227, H274, E277, N294, and E425) (N2 numbering system). Since the active sites of NA are commonly conserved in influenza A and B viruses, these sites are selected as the target of NA inhibitors, such as oseltamivir, zanamivir, and peramivir. Accordingly, these inhibitors bind to the influenza viral NA active site and block the activity of the enzyme, making the influenza virus unable to release from its host cell and infect the adjacent cells [28]. However, mutations within the framework or catalytic sites were reported, which leads to a reduction in binding affinity, lowering the efficacy of the inhibitors among influenza A viruses [29].

The H275Y substitution in the H1N1 subtype of influenza virus A is the most common mutation conferring oseltamivir resistance [43], while frequent amino acid substitutions, E119V and R292K, were found predominantly in the influenza A/H3N2 virus [44]. In Indonesia, two oseltamivir-resistant influenza A/H1N1pdm viruses with H275Y substitution had been found in untreated patients with ILI symptoms [45]. On the contrary, there were no reports found concerning the influenza A/H3N2 subtype with mutations related to oseltamivir resistance.

The occurrence of NA mutations associated with antiviral resistance is becoming a global concern. After four decades, the previous generation of antiviral, the adamantanes or M2 ion channel inhibitors, was no longer recommended due to the emergence of resistant influenza strains harbouring S31N mutations at the M2 protein of the influenza virus [46]. The resistant influenza viruses then spread globally and became a major clinical and public health issue. With an emphasis on the emergence of antiviral drug resistance, continuing the characterization of influenza viruses constitutes an important basis for the control of influenza infection.

The influenza virus by nature is heterogeneous, with a high level of mutation, meaning it undergoes continuous changes through antigenic drift and shift mechanisms. The high mutation rates of RNA viruses, caused by an error-prone RNA-dependent RNA polymerase, and the segmented structures of the influenza virus genome, play roles in the genetic diversity of influenza viruses [41]. Based on these characteristics, previous influenza vaccine strains may not be effective against current circulating influenza strains. Pilgrims travelled to KSA need to be vaccinated as they are at high risk. Unfortunately, no influenza vaccination data were available in this study. More studies are needed to understand the epidemiology of influenza at Hajj, particularly by assessing the efficacy of the influenza vaccine against laboratory-proven influenza in pilgrims.

Together with influenza immunization programs for high-risk groups, antiviral agents can play a major role in the control of seasonal influenza epidemics and may also provide prophylactic and therapeutic benefits during an eventual pandemic. The continuous monitoring of the emergence of drug-resistant strains among circulating influenza viruses and antigenic changes is crucial, especially during mass-gathering events such as Hajj and Umrah where the possibility of air-borne pathogens to be transmitted is high.

## 5. Conclusions

This study describes the distribution of influenza viruses, as well as the genetic characteristics patterns of the influenza A/H3N2 virus in Indonesian Hajj and Umrah pilgrims, from 2013 to 2014. The phylogenetic analysis highlights the dynamics of influenza A/H3N2 virus mutations across time. The study also demonstrates the importance of enhancing quarantine and performing routine genome surveillance towards the incoming Hajj and Umrah pilgrims, particularly those with respiratory disease symptoms.

## Figures and Tables

**Table 1:** Distribution of subtypes of influenza viruses isolated from Hajj and Umrah patients.

Demography	Total	%	Influenza A			Influenza B	Influenza A and B	Negative
			H1pdm	H3	Non H1/H3/H5			
Sex								
Male	126	50.2	29	5	1	15	1	75
Female	125	49.8	22	10	3	14	0	76
Age (y.o.)								
≤50	65	25.9	11	3	3	6	0	42
>50	176	70.1	38	12	1	23	0	102
No Data	10	4.0	2	0	0	0	1	7
Origin								
Sumatra	67	26.7	14	3	0	13	1	36
Java	133	53.0	24	10	4	14	0	81
Bali	11	4.4	3	0	0	0	0	8
Sulawesi	3	1.2	1	0	0	1	0	1
Kalimantan	37	14.7	9	2	0	1	0	25

**Table 2:** Amino acid changes in HA protein of Indonesian H3N2 virus.

AA Position *	AA 2013–2015 Vaccine Strain (A/Texas/50/2012)	AA Sample (n)	Association with Antigenicity
3	L	I (2)	no association [23,24]
10	T	M (2)	no association [23,24]
128	N	A (4); T (4)	antigenic B site [25]
138	A	S (2)	antigenic site A [25]
142	R	G (5)	antigenic site A [25]
144	N	S (2)	antigenic site A, RBD [23,24]
145	N	S (8)	antigenic site A, RBD [23]
157	L	S (2)	antigenic site B [25]
159	F	S (2); Y (2)	antigenic site B [23,25]
160	K	T (2)	antigenic site B [25]
186	V	G (8)	antigenic B, RBD [26]
198	P	S (8)	antigenic site B [25]
219	F	S (8)	antigenic site D [27]
225	N	D (4)	RBD [26]
311	G	H (2)	no association [24]
347	V	M (2)	no association [24]
160	K	T (2)	antigenic site B [25]
186	V	G (8)	antigenic B, RBD [26]
198	P	S (8)	antigenic site B [25]
219	F	S (8)	antigenic site D [27]
489	D	N (4)	no association [24]

AA: amino acid, RBD: receptor-binding domain; * based on H3 numbering system.

## Data Availability

Sequence data obtained from this study are available in the Global Initiative on Sharing All Influenza Data (GISAID) with accession number EPI2274939-EPI2274954.

## Supplementary Materials

The following supporting information can be downloaded at: https://www.mdpi.com/article/10.3390/life13051100/s1, Figure S1: Phylogenetic tree of HA gene of H3N2 virus; Figure S2: Phylogenetic tree of NA gene of H3N2 virus.
