# Supplementary material for: Molecular Characterization of Influenza A/H3N2 Virus Isolated from Indonesian Hajj and Umrah Pilgrims 2013 to 2014
Source: Life (Basel). 2023 Apr 27;13(5):1100. doi: 10.3390/life13051100 (PMC10221221; doi:10.3390/life13051100)

Supplementary data

Molecular characterization of influenza A/H3N2 virus isolated from Indonesian Hajj and Umrah pilgrims 2013 to 2014

Agustiningsih Agustiningsih[1*], Irene Lorinda Indalao[2], Krisnanur A Pangesti[2], Caecilia H C Sukowati[1,3], Ririn Ramadhany[2]

1 Eijkman Research Center for Molecular Biology, National Research and Innovation Agency of Indonesia (BRIN), B.J. Habibie Building, Jl. M.H. Thamrin No. 8, Jakarta Pusat, DKI Jakarta 10340, Indonesia

2 Ministry of Health of the Republic of Indonesia, Jl. H.R. Rasuna Said Blok X.5 Kav. 4-9, Jakarta Selatan, DKI Jakarta 12950, Indonesia

3 Fondazione Italiana Fegato ONLUS, AREA Science Park, Basovizza, Trieste 34049, Italy

* Correspondence: agustiningsih@brin.go.id

Figure S1. Phylogenetic tree of HA gene of H3N2 virus. Only bootstrap values above 70 are shown.

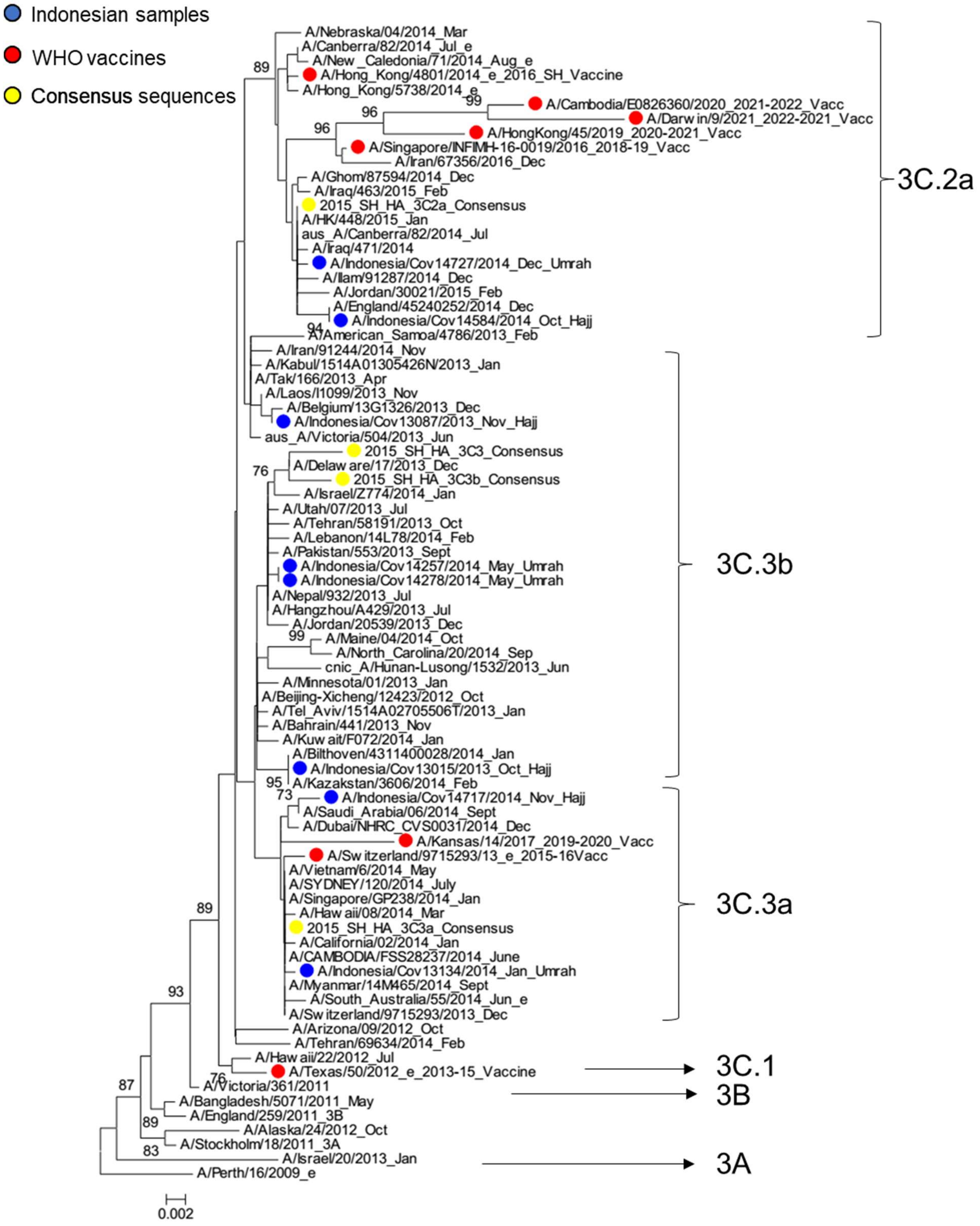

Figure S2. Phylogenetic tree of NA gene of H3N2 virus. Only bootstrap values above 70 are shown.

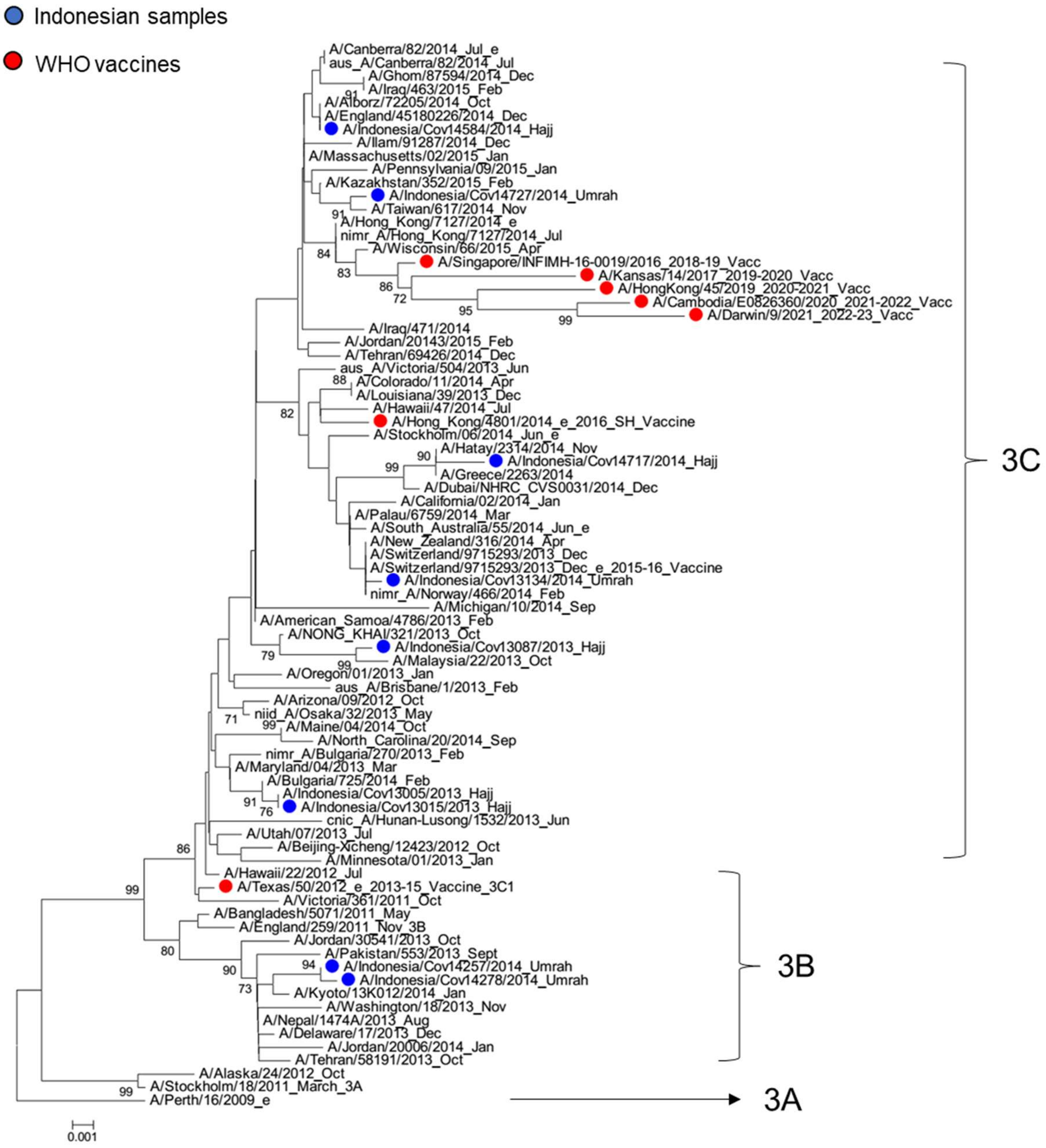

Supplement: Supplementary file 1 [file life-13-01100-s001.zip › life-2268811-supplementary.pdf]
